# A Species delimitation approach to uncover cryptic species in the South American fire ant decapitating flies (Diptera: Phoridae: *Pseudacteon*)

**DOI:** 10.1371/journal.pone.0236086

**Published:** 2020-07-17

**Authors:** Andrés F. Sánchez-Restrepo, Lucila Chifflet, Viviana Andrea Confalonieri, Neil D. Tsutsui, Marcos Antônio Pesquero, Luis Antonio Calcaterra

**Affiliations:** 1 Fundación para el Estudio de Especies Invasivas (FuEDEI), Hurlingham, Buenos Aires, Argentina; 2 Departamento de Ecología, Genética y Evolución, Facultad de Ciencias Exactas y Naturales, Universidad de Buenos Aires (UBA), Instituto de Ecología Genética y Evolución de Buenos Aires (IEGEBA; UBA-CONICET), Buenos Aires, Argentina; 3 Consejo Nacional de Investigaciones Científicas y Técnicas (CONICET), Buenos Aires, Argentina; 4 Museo Argentino de Ciencias Naturales “Bernardino Rivadavia”, Buenos Aires, Argentina; 5 Department of Environmental Science, Policy, and Management, University of California, Berkeley, California, United States of America; 6 Programa de Pós-Graduação em Ambiente e Sociedade (PPGAS), Universidade Estadual de Goiás, Morrinhos, Brasil; National Cheng Kung University, TAIWAN

## Abstract

South American fire ant decapitating flies in the genus *Pseudacteon* (Diptera: Phoridae) are potential biocontrol agents of the invasive fire ants *Solenopsis invicta* and *S*. *richteri* in the United States and other regions of the world due to their high host specificity and the direct and indirect damage to their host ants. Despite their importance and the fact that several flies have already been released in the US, little is known about the genetic variability and phylogenetic relationships of *Pseudacteon* flies parasitizing South American fire ants in the *Solenopsis saevissima* species-group. A species delimitation analysis was conducted using a distance-based method (ABGD) and two tree-based methods (GMYC and mPTP) using COI sequences of 103 specimens belonging to 20 of the 22 *Pseudacteon* species known from southern South America. Additionally, phylogenetic relationships between the already described and new candidate species were inferred using mitochondrial (COI) and nuclear (*wingless*) sequences. The species delimitation analysis suggests that species richness in these flies has been previously underestimated, due to the existence of putative cryptic species within nominal *Pseudacteon obtusus*, *P*. *pradei*, *P*. *tricuspis*, *P*. *cultellatus*, and *P*. *nudicornis*. Geographic distribution and host fire ant species seem to support cryptic lineages, though additional morphological data are needed to corroborate these results. All phylogenetic analyses reveal that South American fire ant decapitating flies are grouped into two main clades, with *Pseudacteon convexicauda* sister and well differentiated relative to these clades. Neither host nor geographic association appeared to be related to the differentiation of these two main clades within South American fire ant decapitating flies. This work provides information that will allow testing whether the putative cryptic phorid fly species show differences in their effectiveness as biocontrol agents against the highly invasive imported fire ants.

## Introduction

Fire ant decapitating flies belong to the genus *Pseudacteon* Coquillett 1907 (Diptera: Phoridae) and derive their common name from their parasitoid life cycle in which their host ants are decapitated after the fly finishes its development inside the ant’s body [1, 2]. During the phorid fly life cycle, the female deposits an egg in the thorax of a living ant worker and, after the egg hatches, the larva migrates into the ant’s head where it consumes the head tissues before pupation. Finally, the adult emerges from the ant’s mouth [2, 3].

The genus *Pseudacteon* is currently comprised of 72 known species distributed worldwide with just over half of them inhabiting the Neotropical region [4]. Most of the New World species are known to be specific parasitoids of fire ants (*Solenopsis* spp.; [5, 6]), but some also parasitize different ant genera, such as *Crematogaster* Lund 1831, *Dorymyrmex* Mayr 1866, *Formica* Linnaeus 1758, *Lasius* Fabricius 1804, *Linepithema* Mayr 1866, *Liometopum* Mayr 1861, *Myrmica* Latreille 1804, *Nylanderia* Emery 1906, *Pseudolasius* Emery 1887, and *Tapinoma* Foerster 1850 [5–8]. At least 22 *Pseudacteon* species are known to parasitize the South American fire ants of the *Solenopsis saevissima* (Smith, F. 1855) species-group and 23 fly species attack fire ants in the *Solenopsis geminata* (Fabricius 1804) species-group [4, 5]. There is no evidence of host switch between *Pseudacteon* species that parasitize these two fire ant groups species despite the overlapping host distribution both in their native and introduced ranges [9, 10]. The only possible host switch was reported for *Pseudacteon fowleri* Pesquero 2000, which is known to parasitize *S*. *saevissima* species-group, but was collected in the range of *S*. *geminata* species-group (in French Guiana and Guyana) [11]. Although these specimens were captured in malaise traps and their host is uncertain, they were placed as part of the ornate oviscapes group defined by [11] due to their morphologically similar genitalia. This group includes *Pseudacteon arcuatus* Borgmeier 1969, *P*. *kungae* Plowes *et al*. 2009, and *P*. *laticarinatus* Plowes *et al*. 2009, which all parasitize fire ants of the *S*. *geminata* species-group. It is possible that the existence of cryptic species underlies this apparent host switch [11].

Fire ant decapitating flies are considered one of the most promising biological control agents of introduced fire ants, *Solenopsis invicta* Buren 1972 and *S*. *richteri* Forel 1909, in North America and other regions of the world [12]. To date, six South American fire ant decapitating fly species have been successfully released by the USDA in the United States as fire ant biocontrol agents: *Pseudacteon curvatus* Borgmeier 1925, *P*. *litoralis* Borgmeier 1925, *P*. *tricuspis* Borgmeier 1925, *P*. *obtusus* Borgmeier 1925, *P*. *nocens* Borgmeier 1926, and *P*. *cultellatus* Borgmeier 1925 [12–14], and other species are being evaluated for future release [12]. However, the success of a biological control program often depends on the choice of appropriate biotypes or geographic races of biocontrol agents [15, 16]. Therefore, proper identification of potential biocontrol agents and accurate knowledge about their phylogenetic relationships are fundamental [11, 17]. Moreover, knowledge of population structure and the relationships between populations of a species can have important implications for the success of biological control [17], particularly when species of interest occur across broad geographic ranges or different habitats [15, 18]. Sometimes, two or more distinct species can be classified under one species name [19]. In these cases, nominal species may harbor cryptic species, which are morphologically similar but can be identified using genetic markers.

Some fire ant decapitating fly species exhibit intraspecific morphological variation across their geographic ranges suggesting the existence of subspecies or cryptic species [8]. For example, *P*. *obtusus* presents variation in the adult body size with a large and a small biotype that form genetically distinct lineages [20], the latter being subsequently described as a new species, *Pseudacteon obtusitus* Plowes, Folgarait, and Gilbert 2015 [21]. Despite the importance of *Pseudacteon* flies in biological control programs for introduced fire ants, knowledge about the phylogenetic relationships between the different species within the genus is scarce [20, 22, 23]. The lack of a phylogenetic hypothesis for fire ant decapitating flies limits their use in biological control because the possible existence of cryptic species can lead to an incorrect choice of a certain biocontrol agent. Adequate species delimitation, enhanced by the use of DNA-based approaches, will help establish accurate species boundaries and enable identification of cryptic or sibling species. The aim of this work is to analyze the species boundaries of the South American fire ant decapitating flies (genus *Pseudacteon*) that parasitize the *S*. *saevissima* species-group in order to elucidate potential new cryptic species within these parasitoid flies and propose phylogenetic hypotheses. Through the use of three species delimitation approaches (a distance-based approach and two tree-based species delimitations) it will be possible to determine if the morphological variability observed in some *Pseudacteon* species corresponds to the presence of cryptic or sibling species, presumably isolated by geographic barriers or host species.

## Materials and methods

### Sample collection and taxonomic coverage

**Fig 1:**
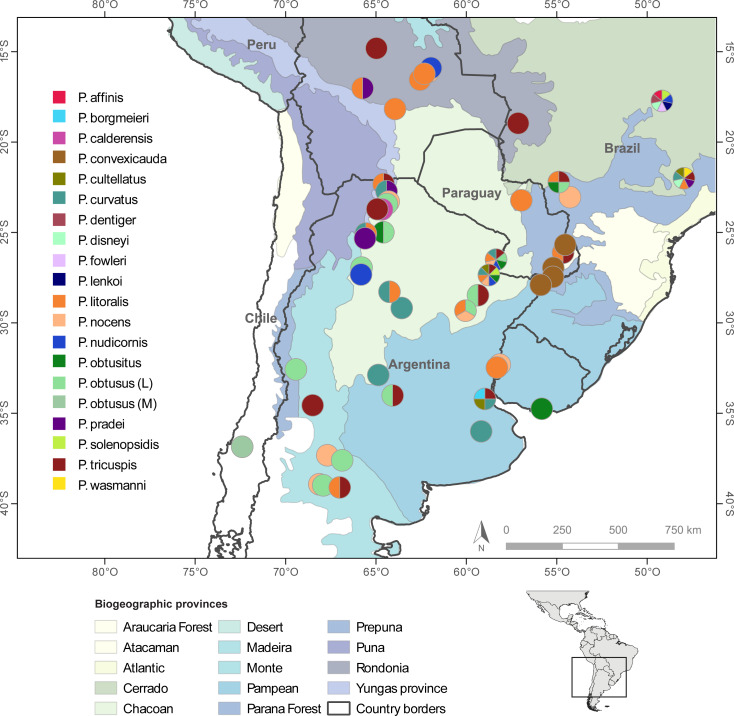
Map showing study area and sampling sites of *Pseudacteon* collected. The colors correspond to the fly species according to their morphological identification. Localities where several species were found are represented as pie charts. Colored regions represent the biogeographic provinces according to Morrone (2014). Map made in QGIS using layers of naturalearthdata.com (administrative) and Löwenberg-Neto 2014 (ecoregions).

Field surveys were conducted across southern South America (Fig 1), between 2002 and 2018. Sample collection consisted of opening and disturbing fire ant nests to attract phorid flies, which were captured using an aspirator during a subsequent ~20-minute period. Specimens were placed in vials with 96% EtOH and stored at 4ºC. Most of the host fire ant species were tentatively identified *in situ* on the basis of the appearance of the colony and later in the laboratory examining major workers under stereomicroscope with available keys [24]. Additionally, some fire ant worker samples were identified based on their cuticular hydrocarbons and molecular analyses [22, 25, 26]. The field studies did not involve endangered or protected species. Though, permissions to collect specimens were obtained in Argentina by Administración de Parques Nacionales and the environment secretariats/ministries of the provinces of Buenos Aires, Santa Fe, Corrientes, Entre Ríos, Formosa, Misiones and Tucumán; in Brazil by Ministerio do Meio Ambiente, Instituto Chico Mendes de Conservação da Biodiversidade–ICMBio; in Bolivia by Museo de Historia Natural Noel Kempff, Santa Cruz de la Sierra; in Uruguay, Paraguay and Chile no permissions were required to collect no endangered species.

A total of 103 individuals belonging to 20 nominal *Pseudacteon* species was sampled from nests of nine fire ant species (Table 1; Fig 1). This richness corresponds to ~90% of all fire ant decapitating fly species known to parasitize fire ants in the *S*. *saevissima* species-group. Samples of *Pseudacteon convexicauda* Borgmeier 1962, collected on nests of the tawny crazy ant *Nylanderia fulva* (Mayr 1862) from Argentina were also included. *P*. *convexicauda* is the only phorid fly species reported using both the tawny crazy ant and the fire ant *S*. *saevissima* as host [1, 27] Morphological identifications of flies were made using the keys provided by [8] and confirmed by comparison to vouchers in the reference collection at the Fundación para el Estudio de Especies Invasivas (FuEDEI; Hurlingham, Buenos Aires), together with more recent species descriptions [21, 28–30]. Vouchers were deposited at FuEDEI reference collection and Museo Argentino de Ciencias Naturales “Bernardino Rivadavia” (MACN).

**Table 1:** Summary of species delimitation methods in relation to the a priori morphological identifications of the South American fire ant decapitating flies (*Pseudacteon*) and its host ant.

Morphological identification	n	Host ant	Species identity from each delimitation method *		
			ABGD	GMYC	mPTP
*P*. *affinis*	2	*S*. *saevissima*	*P*. *affinis*	*P*. *affinis*	*P*. *affinis*
*P*. *borgmeieri*	2	*S*. *richteri*	*P*. *borgmeieri*	*P*. *borgmeieri*	*P*. *borgmeieri*
*P*. *calderensis*	2	*S*. *interrupta*	*P*. *calderensis*	*P*. *calderensis*	*P*. *calderensis*
*P*. *convexicauda*	4	*N*. *fulva*	*P*. *convexicauda*	*P*. *convexicauda*	*P*. *convexicauda*
*P*. *cultellatus*	4	*S*. *invicta*	*P*. *cultellatus* (a)	*P*. *cultellatus* (a)	*P*. *cultellatus* (a)
		*S*. *richteri*			
		*S*. *invicta*	*P*. *cultellatus* (b)	*P*. *cultellatus* (b)	*P*. *cultellatus* (b)
*P*. *curvatus*	10	*S*. *invicta*	*P*. *curvatus*	*P*. *curvatus*	*P*. *curvatus*
		*S*. *interrupta*			
		*S*. *richteri*			
*P*. *dentiger*	2	*S*. *saevissima*	*P*. *dentiger*	*P*. *dentiger*	*P*. *dentiger*
*P*. *disneyi*	2	*S*. *saevissima*	*P*. *disneyi*	*P*. *disneyi*	*P*. *disneyi*
*P*. *fowleri*	1	*S*. *saevissima*	*P*. *fowleri*	*P*. *fowleri*	*P*. *fowleri*
*P*. *lenkoi*	1	*S*. *saevissima*	*P*. *lenkoi*	*P*. *lenkoi*	*P*. *lenkoi*
*P*. *litoralis*	18	*S*. *invicta*	*P*. *litoralis + P*. *nocens*	*P*. *litoralis + P*. *nocens* (in part)	*P*. *litoralis + P*. *nocens*
		*S*. *interrupta*			
*P*. *nocens*	9	*S*. *invicta*			
		*S*. *interrupta*		*P*. *nocens* (a)	
*P*. *notocaudatus*	4	*S*. *interrupta*	*P*. *notocaudatus*	*P*. *notocaudatus*	*P*. *notocaudatus*
*P*. *nudicornis*	6	*S*. *invicta*	*P*. *nudicornis* (a)	*P*. *nudicornis* (a)	*P*. *nudicornis* (a)
		*S*. *interrupta*		*P*. *nudicornis* (c)	*P*. *nudicornis* (c)
		*S*. *saevissima*	*P*. *nudicornis* (b)	*P*. *nudicornis* (b)	*P*. *nudicornis* (b)
*P*. *obtusitus*	5	*S*. *invicta*	*P*. *obtusitus + P*. *obtusus L* (c) (in part)	*P*. *obtusitus + P*. *obtusus L* (c) (in part)	*P*. *obtusitus + P*. *obtusus L* (c) (in part)
*P*. *obtusus (L)*	11	*S*. *invicta*			
		*S*. *macdonaghi*			
		*S*. *interrupta*	*P*. *obtusus* L (a)	*P*. *obtusus* L (a)	*P*. *obtusus* L (a)
		*S*. *richteri*	*P*. *obtusus* L (b)	*P*. *obtusus* L (b)	*P*. *obtusus* L (b)
*P*. *obtusus (M)*	1	*S*. *gayi*	*P*. *obtusus* M	*P*. *obtusus* M	*P*. *obtusus* M
*P*. *pradei*	2	*S*. *interrupta*	*P*. *pradei* (b)	*P*. *pradei* (b)	*P*. *pradei* (b)
		*S*. *invicta*	*P*. *pradei* (c)	*P*. *pradei* (c)	*P*. *pradei* (c)
*P*. *solenopsidis*	2	*S*. *invicta*	*P*. *solenopsidis*	*P*. *solenopsidis*	*P*. *solenopsidis* (a)
		*S*. *saevissima*			*P*. *solenopsidis* (b)
*P*. *tricuspis*	14	*S*. *invicta*	*P*. *tricuspis* (a)	*P*. *tricuspis* (a)	*P*. *tricuspis* (a)
		*S*. *interrupta*			
		*S*. *richteri*			
		*S*. *saevissima*			
		*S*. *invicta*	*P*. *tricuspis* (b)	*P*. *tricuspis* (b)	*P*. *tricuspis* (b)
*P*. *wasmanni*	1	*S*. *invicta*	*P*. *wasmanni*	*P*. *wasmanni*	*P*. *wasmanni*

n = total number of specimens, ABGD = Automatic Barcode Gap Discovery, GMYC = Generalized Mixed Yule Coalescent method, mPTP = multi rate Poisson Tree Processes.

* In those cases where a morphology identified species or biotype was split into several a letter in parenthesis is assigned to distinguish them. The “+” sing means that two species or biotypes (or parts of them) were retrieved as a single unit.

### DNA amplification and sequencing

Total genomic DNA was extracted from one specimen per host nest using the REDExtract-N-Amp^TM^ Tissue PCR Kit (Sigma-Aldrich, St. Louis, MO, USA). Fragments of the *cytochrome oxidase subunit I* (COI) (mitochondrial) and *wingless* (*wg*) (nuclear) genes were amplified by PCR (Polymerase Chain Reaction) using the primers C1-J-2183 (alias Jerry) and TL2-N-3014 (alias Pat) for COI [31], and LepWG1 and LepWG2a for *wg* [32] (details in S1 Table). PCR reactions were carried out in a 20 μl volume reaction containing: 4 μl genomic DNA extract (50–100 ng), 10 μl REDExtract-N-Amp™ PCR ReadyMix, 0.5 μl of each primer (10 μM), and 5 μl of distilled water. Thermal cycling conditions were as follows: denaturation at 95°C for 3 min, then 37 cycles of denaturation at 94°C for 1 min, annealing at 50°C for 1 min, and extension at 74°C for 1 min, followed by a final extension at 74°C for 10 min. PCR products were visualized on a 1% agarose gel stained with GelRed (Biotium, Hayward, CA, USA) and 15 μl of PCR product were purified using a mixture of 0.5μl (10u) FastAP (Thermosensitive Alkaline Phosphatase), 1 *μl* (1u) ExoI (exonuclease I) incubated at 37°C for 15 min, stopping the reaction by heating the mixture at 85°C for 15 min. Most of the purified PCR products were sequenced at the sequencing and genotyping unit of the Faculty of Exact and Natural Sciences of the University of Buenos Aires (FCEyN, UBA, Buenos Aires, Argentina), using a 3130-XL Automatic Sequencer (Applied Biosystems), and some of the samples were sequenced at the UC Berkeley DNA Sequencing Facility. A total of 102 COI and 48 *wg* sequences were inspected, trimmed and aligned using Geneious Pro v4.8 (http://www.geneious.com/) and deposited at GenBank (accession numbers MN787931 to MN788076; S5 Table).

### Species delimitation

The traditional phenotype-based species boundaries in South American fire ant decapitating flies was corroborated using three different analyses: a distance-based approach (Automatic Barcode Gap Discovery–ABGD, [33]) and two tree-based species delimitations (Generalized Mixed Yule Coalescent method–GMYC, [34]; and multi rate Poisson Tree Processes–mPTP, [35]). The dataset employed for all methods consisted of COI sequences from 20 putative *Pseudacteon* species and five outgroups (*Anevrina variabilis* (Brues 1908), *Apocephalus paraponerae* Borgmeier 1958, *Argentinomyia longicornis* (Walker 1836), *Drosophila acanthoptera* Wheeler 1949, *Stichillus* Ederlein 1924). GenBank accession numbers of outgroups sequences: GU559934.1, AF217478.1, DQ448549.1, KM270850.1, KF632601.1, GU559945.1. All identical sequences were removed prior analyses, resulting in a 91 COI sequence dataset, including the outgroups. All species delimitation analyses were conducted using a pruned outgroup tree and a complete tree including the outgroups.

The ABGD assumes that the intraspecific divergence is smaller than the interspecific divergence [33]. In other words, it infers the barcode gap by recursively finding the first significant gap beyond limit for intraspecific divergence and uses it to partition the data until there is no further partitioning. A Kimura two parameter (K2P) distance matrix was generated in Mega v.7 [36] as input and analyzed using the online ABGD service (http://wwwabi.snv.jussieu.fr/public/abgd/), testing different relative gap width (*X*) values between 0.8 and 1.0 and intraspecific divergence (*P*) values between 0.001 (0.1%) and 0.1 (10%).

The tree-based methods employ a coalescent framework in order to independently identify evolving lineages without gene flow, each representing a putative species [37]. They can be performed using a single marker or a multi–locus dataset and are used to establish a threshold that show the separation of intraspecific population substructure from interspecific divergence, and therefore identify those groups that may be candidate species [38].

The GMYC method [34, 39, 40] employs a coalescent model of intraspecific branching combined with a Yule model for interspecific branching, using an inferred single-gene topology to reconstruct single locus gene trees and estimate a statistical measure of confidence for the inferred boundaries [38]. GMYC is relatively robust when only single-locus data are available [40]. As input, an ultrametric tree generated in BEAST v.1.8.2 [41] under a strict molecular clock and a coalescent constant size tree prior was used. A Markov Chain Monte Carlo (MCMC) of 20 million generations, sampling every 1000 generations was conducted in two independent runs. The effective sample size (ESS) for each parameter estimated was checked to be > 200 and the first 10% of the trees were discarded as the burn-in period using Tracer v.1.6 [42]. The maximum credibility clade tree was found using TreeAnnotator [41] with all options set to default. GMYC analyses were performed on the R platform (R Development Core Team, 2016) using the latest implementation of the “splits” package version 1.0–19 [43], employing a single and multiple threshold with a confidence interval falling within ± 2 log likelihood units around the ML estimate. The point of highest likelihood of this mixed model (threshold) was interpreted as the species boundary [34].

The third species delimitation approach was implemented using a Poisson tree process (PTP), which models the speciation using the number of substitutions to infer putative species boundaries on a given phylogenetic input tree [35]. It assumes that the number of substitutions between species is significantly higher than the number of substitutions within species [35]. Here, we used the multi-rate PTP (mPTP) as proposed by [44], which incorporates the potential divergence in intraspecific diversity to the PTP and implements a fast method to compute the maximum likelihood delimitation from an inferred phylogenetic tree of the samples. Contrary to GMYC, mPTP only needs a simple phylogenetic tree rather than an ultrametric tree [38, 44]. Hence, we estimated a maximum likelihood gene tree using RAxML v.8.2.10 [45], spending the GTRGAMMA substitution model and bootstrap support values were estimated from 1000 replicates. Analyses were run for the single and multiple thresholds using the command line of mPTP.

### Phylogenetic analyses

To more completely understand the phylogenetic relationships between the South American *Pseudacteon* species parasitizing fire ants, we analyzed a subset of the COI sequences plus additional sequences of the nuclear gene *wg* from 27 samples that represents the best supported lineages/species identified during the species delimitation analyses. The dataset (COI + *wg*) was analyzed in Bayesian inference framework as implemented in BEAST v.2.5.1 [46]. We assumed a Yule speciation model with constant population size model and strict molecular clock. The dataset was partitioned per marker and substitution models were selected for each -COI and *wg-* partitions using bModelTest [47]; this BEAST2 package allows exploration of the substitution model space while simultaneously estimating model parameters and the phylogeny. To estimate the divergence times a Bayesian approach was carried out in BEAST v.2 .5.1 [46]. The tree parameters were linked through the partitions using a strict clock and Yule model tree priors. As node age calibration points two phorid fossils were used: †*Anevrina oligocoenica* Brues 1939, known from Eocene/Oligocene Baltic amber (37.2–33.9 Ma; [48, 49]) and †*Apocephalus succineus* Brown 2000, known from Oligocene/Miocene Dominican amber (15–20 Ma; [50]). The analysis was performed in the CIPRES Science Gateway v.3.3 [51] by running two independent replicates of a MCMC of 40 million generations, sampling trees every 1000 generations. Convergence was evaluated by accessing log files in Tracer v1.6 [42]. Both replicates were combined in LogCombiner, using a burn-in of 25% for each run. Finally, a calibrated, in million years (Ma), maximum clade credibility tree was generated in TreeAnnotator [46].

Additionally, to establish the congruence of the relationships found in the maximum credibility tree further phylogenetic reconstructions were assessed by maximum parsimony (MP) and maximum likelihood (ML) methods. MP analysis was carried out with TNT v.1.5 [52] employing heuristic searches with ratchet, tree-drifting, sectorial and tree-fusing, using default settings and best score hits of 10. Support for individual nodes was assessed by bootstrap resampling (1000 replicates). The ML analysis was conducted with RAxML v.8.2.10 [45], using the GTRGAMMA substitution model, with two partitions (COI and *wg*), and bootstrap support values were estimated from 1000 replicates.

## Results

### Species delimitation

**Fig 2:**
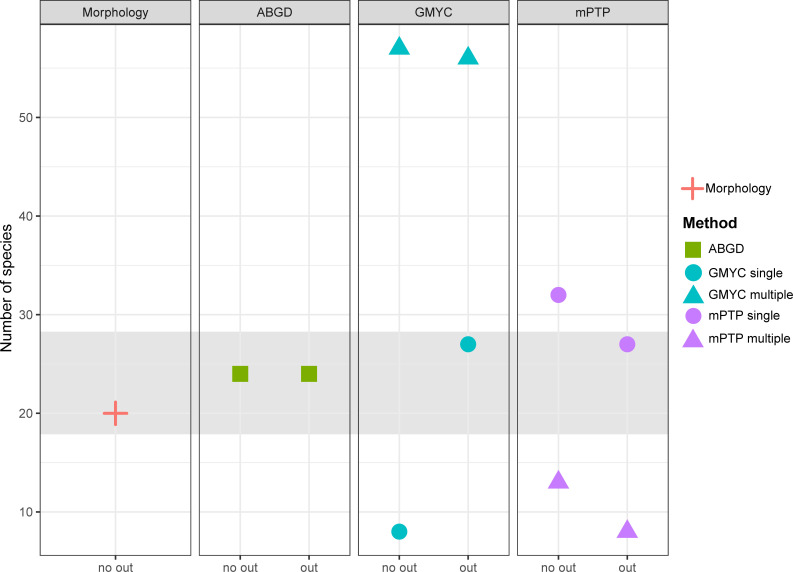
Summary of number of species lineages obtained with ABGD, GMYC and mPTP species delimitation methods, and tested combinations. GMYC and mPTP results are shown with different thresholds (single = circles, multiple = triangles) and for all methods including (out) or excluding (no out) the outgroups. The number of morphologically identified species is shown as a reference.

The COI alignment consisted of 91 sequences of 699 base pairs (bp) and 316 segregating (polymorphic) sites. In most of the ABGD, GMYC and mPTP analyses, the number of lineages found was higher than those identified by morphology (Fig 2). The analyses pruning the outgroups were less consistent and tended to substantially increase or decrease the number of delimited lineages, except for ABGD (Table 1; Fig 2). The differences between the single and multiple thresholds were very variable: multiple GMYC overestimated the number of lineages, while multiple mPTP underestimated it. Despite these differences, species number estimation including the outgroups and single thresholds were closer to the number of species morphologically identified and also showed less variation between each method (ABGD = 24, GMYC = 27, mPTP = 27). The difference in the number of species estimated with GMYC using single and multiple thresholds was substantially wide in comparison to mPTP.

The topologies generated during the delimitation analyses using the COI dataset, for the distance method (ABGD; S1 Fig and S2 Table) and the input trees for the GMYC (S2 and S3 Figs; S3 Table) and mPTP (S4 and S5 Figs; S4 Table) show *P*. *convexicauda* as a well-defined sister clade to the rest of the *Pseudacteon* species studied. Also, all the topologies recover two well-supported clades within the South American fire ant decapitating flies: Clade A comprised of *Pseudacteon nudicornis* Borgmeier 1925, *P*. *borgmeieri* Schmitz 1923, *P*. *wasmanni* (Schmitz 1914), *P*. *affinis* Borgmeier 1962, *P*. *cultellatus*, *Pseudacteon pradei* Borgmeier 1925, *Pseudacteon notocaudatus* Plowes *et al*. 2015, and *Pseudacteon disneyi* Pesquero 2000, and Clade B includes *Pseudacteon solenopsidis* (Schmitz 1914), *P*. *dentiger* Borgmeier 1962, *P*. *fowleri*, *Pseudacteon lenkoi* Borgmeier & Prado 1975, *P*. *tricuspis*, *Pseudacteon calderensis* Calcaterra 2007, *P*. *nocens*, *P*. *litoralis*, *P*. *curvatus*, *P*. *obtusus* (large (L) and medium size (M) biotypes), and *P*. *obtusitus* (formerly small biotype of *P*. *obtusus*). No evident host or geographic association was found to be related to the differentiation of these two main clades. Three species *P*. *nudicornis*, *P*. *solenopsidis*, and *P*. *obtusus* (M biotype) varied their position in the phylogeny between for the GMYC and mPTP input trees (S2, S3, S4 and S5 Figs). However, no variation or change of species from clade A to clade B and vice versa was observed in the input trees.

**Fig 3:**
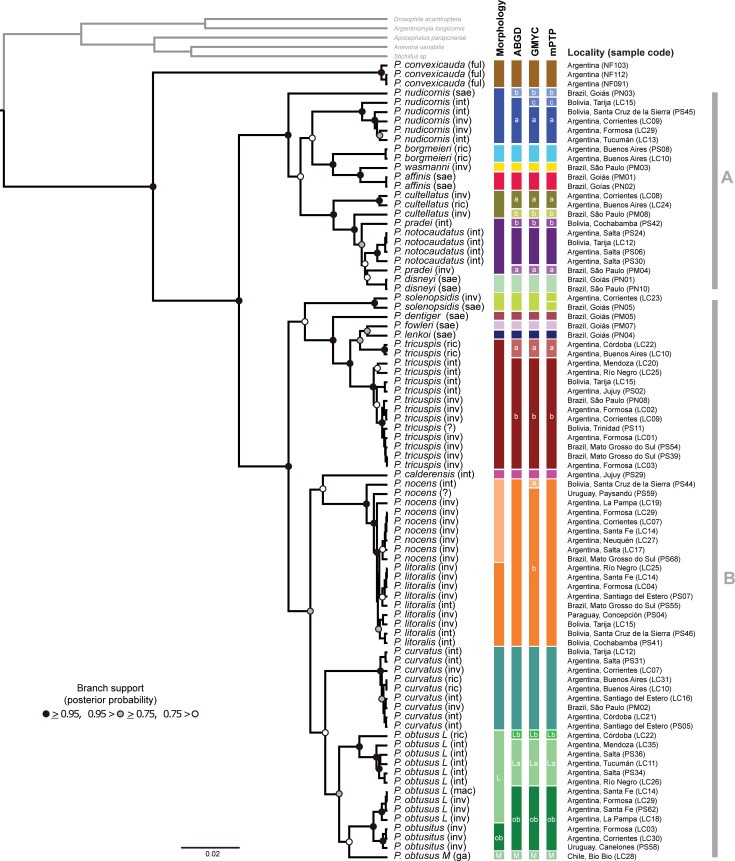
Summary of the three species delimitation analyses (ABGD, GMYC and mPTP) using COI. Bars at the tips of the tree indicate the corresponding result for each species delimitation method, the first corresponding to the morphological identification, letter in bars is assigned in those cases where a species or biotype was split (same as used in Table 1 and Fig 4). The topology shown corresponds to the Bayesian ultrametric tree obtained in BEAST, based in COI only. Parentheses at the end of the terminal names represent the ant nest (host) where the specimen was collected (inv = *Solenopsis invicta*, int = *S*. *interrupta*, ga = *S*. *gayi*, mac = *S*. *macdonaghi*, ric = *S*. *richteri*, sae = *S*. *saevissima*, ful = *Nylanderia fulva*,? = host not recorded). Vertical grey bars indicate the two main clades found.

All methods split each one of the following species: *P*. *cultellatus*, *P*. *nudicornis*, *P*. *obtusus*, *P*. *pradei* and *P*. *tricuspis* into two or three different clades (Table 1) and group *P*. *litoralis* and *P*. *nocens* into a single species (Fig 3). The *P*. *nocens* (PS44) from Bolivia was the most differentiated lineage of the group (in GMYC appears in a different clade) and could be related to an Amazon (basin) lineage of the genus (not included in this study). Other two lineages of *P*. *nocens* were found: one in Uruguay and another one in La Pampa, Argentina. The remaining specimens constitute a clade with two lineages, one composed by *P*. *nocens* from Brazil and Argentina and the other one composed of all the individuals morphologically identified as *P*. *litoralis*. The latter comprises a group of populations from Brazil and Argentina and another one from Paraguay and Bolivia.

**Fig 4:**
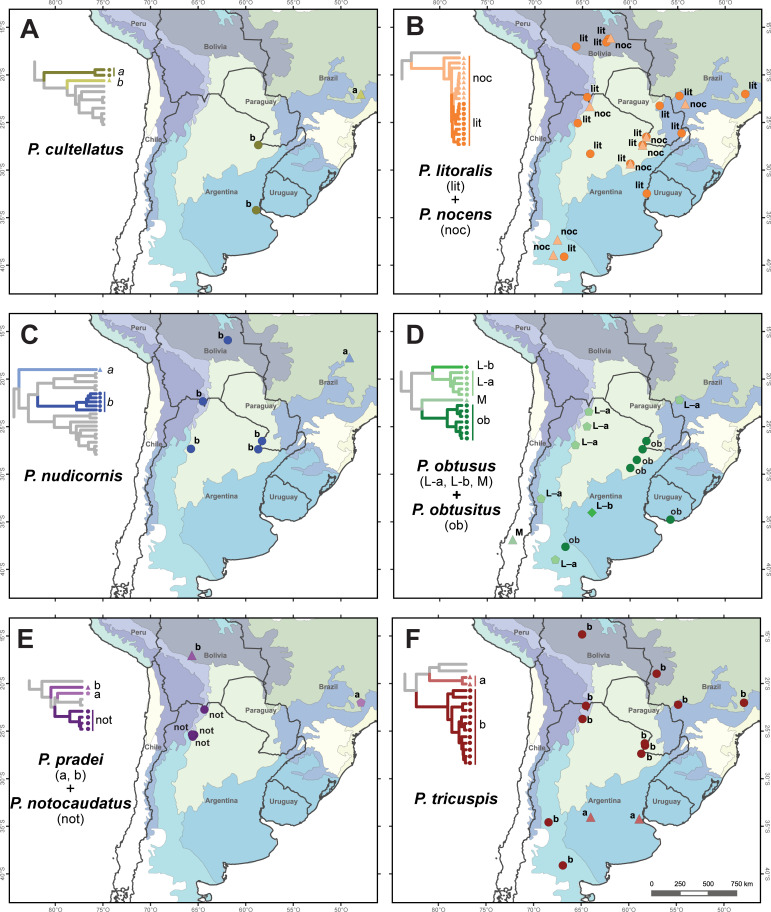
Geographic distribution of South American fire ant decapitating fly species constituted by multiple genetic lineages as shown in the species delimitation analysis. In each map the tree corresponds to the result of the species delimitation analyses (Fig 3), colors and point shapes are used to distinguish each genetic lineage. A) *Pseudacteon cultellatus*, B) *P*. *litoralis* + *P*. *nocens*, C) *P*. *nudicornis*, D) *P*. *obtusus* complex, E) *P*. *pradei*, and F) *P*. *tricuspis*. Colored regions represent the biogeographic provinces according to Morrone (2014), see Fig 1 for conventions. Maps made in QGIS using layers of naturalearthdata.com (administrative) and Löwenberg-Neto 2014 (ecoregions).

In all COI topologies *P*. *cultellatus* resolves as paraphyletic, being composed of two different clades: a specimen found in a *S*. *invicta* colony in Brazil (Fig 4A) occurs within a sister clade to *P*. *pradei* + *P*. *notocaudatus* + *P*. *disneyi* clade. The remaining *P*. *cultellatus*, from more southern localities in Argentina and found attacking *S*. *invicta* and *S*. *richteri* colonies, constitute a well-defined clade sister to *P*. *cultellatus* (from Brazil) + *P*. *notocaudatus* + *P*. *pradei* + *P*. *disneyi*. Also, in this group, *P*. *pradei* appears into two different lineages, also paraphyletic, with one clade (from Brazil, parasitizing *S*. *invicta*) being sister of *P*. *disneyi* (Fig 4E), and another clade attacking *Solenopsis interrupta* Santschi 1916 in Bolivia (Fig 4E).

According to all species delimitation analyses, *P*. *nudicornis* splits into two (ABGD) or three (GMYC and mPTP) putative species. In all analyses, the specimen from Goiás (Brazil) is placed as a distant lineage separated from all the other *P*. *nudicornis* (Fig 4C). The remaining specimens are grouped into either one (ABGD) or two (GMYC and mPTP) lineages distributed across Argentina and central Bolivia (Fig 3). The specimen from Brazil is also differentiated in relation to its host affinity, as it was found parasitizing *S*. *saevissima* (Fig 4C). All the specimens of *P*. *nudicornis* are sister to the clade composed by *P*. *affinis*, *P*. *wasmanni*, and *P*. *borgmeieri*, except the specimen from Brazil (*P*. *nudicornis* “b”), which appears to be a well-supported different species (Figs 3 and 4C).

*Pseudacteon obtusus* + *P*. *obtusitus* split into four well differentiated clades according to all species delimitation analyses (Fig 3). One clade includes *P*. *obtusitus* + *P*. *obtusus* (L) that uses *S*. *invicta* and *Solenopsis macdonaghi* Santschi 1916 as hosts, and are distributed in northeastern Argentina; another clade is composed of by *P*. *obtusus* (M) from Chile that parasitizes *Solenopsis gayi* (Spinola 1851) (Fig 3); another clade is composed of an individual from Córdoba province in central Argentina (Fig 4D) which uses *S*. *richteri* as host; and finally the fourth clade includes *P*. *obtusus* (L) specimens that use *S*. *interrupta* as host and are mainly distributed in the Monte and Yungas ecoregions in western Argentina.

Most specimens of *P*. *tricuspis* form a well-defined clade (that parasitizes *S*. *invicta*, *S*. *interrupta*, and *S*. *saevissima*). However, two specimens that parasitize *S*. *richteri* in La Pampa ecoregion constitute another well-defined clade, closely related to *P*. *lenkoi* and *P*. *fowleri* (Figs 3 and 4F).

### Phylogenetic relationships

**Fig 5:**
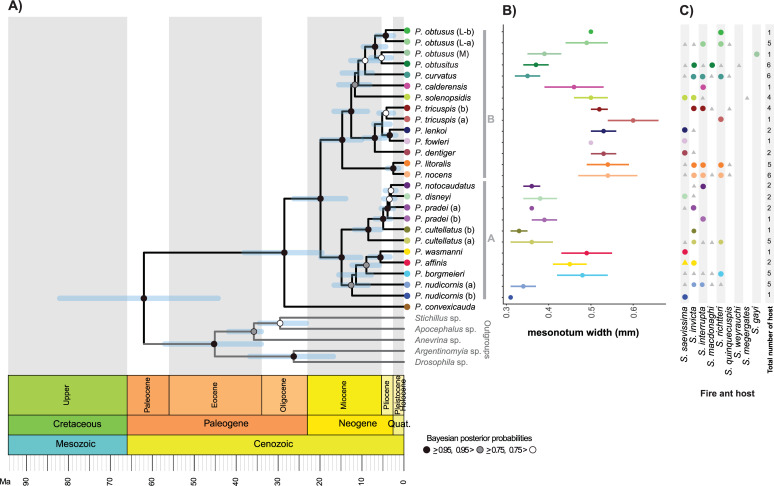
Phylogenetic analysis of the South American fire ant decapitating flies (*Pseudacteon* spp.). A) Maximum clade credibility tree generated in BEAST2 using a COI + *wg* dataset for each species/candidate-species of *Pseudacteon* that parasitizes fire ants of *S*. *saevissima* species-group. The tree has branch lengths in time units (Ma) and showed on a stratigraphic time scale. The uncertainty (95% highest posterior density intervals) in the node ages is indicated with blue bars. B) Mean mesonotum width (in mm) for each species in the tree, the bars indicate the standard error. C) Fire ant presumptive host for each of the *Pseudacteon* species studied, circles correspond to our data and grey triangles are the hosts reported by [4].

Both mitochondrial and nuclear markers were variable and informative for detecting variability within the species studied. The *wg* alignment consisted of 51 sequences of 630 bp and 101 segregating (polymorphic) sites. All studied *Pseudacteon* species (26) were recovered as a single group, with the only exception of *P*. *convexicauda* that was recovered as sister to all the other species (Fig 5). The consensus topologies obtained with each phylogenetic reconstruction method were very similar and congruent for most relationships (results for each method and marker can be found in supporting information S6 Fig). However, *P*. *calderensis* and *P*. *solenopsidis* differed in their phylogenetic position among the different methods. Similar, to the COI trees (Fig 3), the COI-*wg* species tree revealed two main well supported clades (Fig 5). Divergence time estimation suggests that the origin of the South American *Pseudacteon* fly species occurred approximately during the Oligocene / Miocene (28.51 Ma, 95% HPD 19.6–38.1) and the split between the two major clades A and B occurred during the early Miocene (19.89 Ma, 95% HPD 13.8–26.3). Moreover, the analysis suggests that most of the species diverged during the early Pliocene (~4 Ma).

## Discussion

Most previous studies on South American fire ant decapitating flies have been focused on understanding their host specificity [10, 53, 54], behavior and activity patterns [1, 23, 55], or geographical distribution [22, 25, 56]. The present study is first to delineate species boundaries at molecular level and reconstruct putative phylogenetic relationships among the South American fire ant decapitating flies. We found that the 20 *Pseudacteon* species studied in this work that parasitize fire ants of the *S*. *saevissima* species-group belong in fact to at least 24 to 27 species. Alongside the morphological evidence, our study suggests that the number of South American fire ant decapitating fly species (around 27–30 species considering those not included in our study: *Pseudacteon comatus* Borgmeier 1925, *Pseudacteon conicornis* Borgmeier 1962, *Pseudacteon bulbosus* Brown *et al*. 2003) has been previously underestimated (20 to 22 species according to [56] and [4]) and some taxonomic changes to maintain a reliable classification system are needed.

### Species delimitation

GMYC, mPTP and ABGD results reveal some putative cryptic groups, showing that the genus *Pseudacteon* may be more diverse than what traditional morphology-based taxonomy suggests. One of the limitations of the approach employed here is related to the use of a single-locus dataset (e.g. the possible discordance between species trees and gene trees [57] or the difficulty of detecting independent evolution compared with multi-locus approaches [58]). However, the COI marker selected corresponds to a widely barcode region used for molecular taxonomy and systematic of different organisms. This barcode region has demonstrated to be a very efficient marker for species identification as it has good discrimination power for most animal groups [59] and the advantage of presenting low interspecific variability and high divergence between species. Also, our approach includes the comparison of different types of species delimitation methods and parameters in order to avoid the over- or sub-estimation of the candidate species by using only one method, as has been shown by different studies [35, 60]. In other words, the application of multiple methods allows to evaluate which one is better in relation to the phenotype-base identification (as seen in Fig 2). The distance method used here, commonly used in barcode analytical frameworks, though being fast and simple, have the disadvantage of being independent of tree topology and rely on the sliding window applied to the barcode gap. In relation to the tree-based methods the GMYC performs better when the population sizes tend to be small and divergence times between species tend to be high [40] while the mPTP was proposed as a better and fasted alternative to the former methods [44], but with similar limitations when used with single-locus data. The differences in using single or multiple thresholds is associated by the mean population size relative to divergence times, as this has been showed to affects the accuracy of the tree-based methods [40, 61]. For this reason, and similar to our results, other studies have found that single-threshold version usually outperforms the multiple-threshold approach [39, 40]. In summary, although the appropriate uses of species-delimitation methods have been under debate [62], analytical protocols using single-locus data can serve as a starting point to corroborate traditional phenotype based species boundaries in understudied groups and identify species that have previously been hidden within nominal species [38]. Furthermore, the use of complementary information (e.g. geographical, ecological, behavioral, and morphological) enables more robust taxonomic decisions.

The species delimitation methods that used more conservative (single) thresholds resulted in closer estimates to the number of morphological delimited species based on morphological characters as compared to multiple thresholds. This may be due to differences among the algorithms, the guide tree used and/or to sampling representation, as mentioned in the literature [35, 38]. Also, the inclusion of outgroups differentially affected the estimated number of species, with GMYC being more sensitive to this inclusion than mPTP, probably as a consequence of the guide tree used. While GMYC identifies the transition points between inter and intra-species branching rates on an ultrametric tree, mPTP directly uses the number of substitutions [35]. Despite the difficulties of using only one marker to delimit these flies, the use of three different approaches carried out, including the outgroups, enabled us to establish potential boundaries among the South American *Pseudacteon* fly species that parasitize fire ants in the *S*. *saevissima* species-group. Also, our approach using multiple species delimitation methods allowed us to recognize possible spurious or overestimated results.

### Species boundaries

This study suggests that some nominal species among the South American fire ant decapitating flies with broad geographic distributions, are actually composed by the nominal and one or more cryptic species (e.g. *P*. *tricupsis*, *P*. *nudicornis*, and *P*. *obtusus* complex; Fig 4C, 4D and 4F). Given the wide distribution of these species they tend to overlap with the distribution of multiple species of fire ants of the *S*. *saevissima* species-group [56]. The fact that one species of fire ant decapitating fly can attack multiple species of fire ants along its range of distribution has already been mentioned [22, 25, 63]. It is known that that distribution of these parasitoid flies is mainly determined by its host specificity [64], that those with wider geographical ranges which often attack multiple species of fire ants (usually, too, with wide ranges) [63] and, in this sense, the broader the ranges seems to be the result of parasitoid fly species using multiple host [56]. But particularly, is also known that in a single location multiple *Pseudacteon* species may coexist and parasitize the same host species [2, 9, 23]. This phenomenon has been explained by the existence of niche partitioning [2, 65], as occurs when, for example, different fly species with different body sizes attack different sizes of host fire ant workers [66], have different pattern of daily activity [23, 67], and/or differentially attack at nest mounds or on foraging trails [65]. This phenomenon could explain the high diversity of flies attacking a single, though diverse, group of ants compared to other ant parasitoids [5]. In other words, these behavioral factors could be keys for the existence of cryptic species, perhaps as a consequence of sympatric speciation.

Some species in our analyses were represented by a single individual (e.g. *P*. *fowleri*, *P*. *lenkoi*, *P*. *wasmanni*, and *P*. *obtusus* (M)) or were reduced to a single sequence during the analyses because these were identical (e.g. *P*. *calderensis* and *P*. *dentiger*). Although, the support for these flies as distinct species is consistent with their morphology, genetic distance, and estimated times of divergence, these results should be taken with caution until a larger number of individuals and populations can be included, or additional morphological and molecular evidence can be analyzed.

Our results confirm that the *P*. *obtusus* species-complex is comprised of different lineages, related to the host fire ant and/or geographic distribution rather than to body size. The phylogenetic relationships within this species-complex were previously analyzed by [20], who found that the large and small biotypes fall into genetically distinct lineages. Later, [21] described the small biotype as *P*. *obtusitus* based on morphological characters. Our study supports the existence of these differences based on the results of the species delimitation methods, but also shows no clear relationship between the large body size and the genetic lineages found in the phylogenetic analysis using COI + *wg* (Fig 5), at least not enough as to differentiate these species. Indeed, eastern Argentine populations of *P*. *obtusus* (L) were considered by all delimitation analyses as the same species as *P*. *obtusitus* (Table 1, Fig 3). Interestingly, these two groups were always found using *S*. *invicta* as a host. The remaining *P*. *obtusus* (L) were split by the delimitation analyses in two putative species (Figs 4D and 5). Also, our results provide evidence (e.g. large genetic distance) that supports that the medium size biotype of *P*. *obtusus* (M) from Chile belongs to a different species, sister of *P*. *obtusitus*. In agreement with this result, the female ovipositor of this biotype is more rounded than the Argentine large biotype and is the only known species of *Pseudacteon* that attacks *S*. *gayi* [22]. Based on these observations, we suggest that *P*. *obtusus* complex needs to be revised and biotypes/lineages need to be redefined taking into account their geographic distribution, morphology, host preferences, and perhaps also body size.

The clade including the nominal species *P*. *cultellatus*, *P*. *pradei*, *P*. *notocaudatus*, and *P*. *disneyi* also shows the existence of cryptic and/or paraphyletic species (Figs 3, 4A and 4E). The specimens of *P*. *pradei* were collected more than 100 km apart (Fig 4E) and differed in their host species, supporting that the two lineages identified possibly constitute different species. The original description of *P*. *pradei* was made based on specimens collected in Petrópolis, Río de Janeiro, Brazil [68], thus the lineage referenced as “*P*. *pradei* a” in Figs 4E and 5 possibly corresponds to the originally described as *P*. *pradei*. Interestingly, closely related to *P*. *pradei* and *P*. *disneyi* is *P*. *notocaudatus* which was discovered in northwestern Argentina and was tentatively named as *P*. cf. *disneyi* [25]

The species delimitation of *P*. *cultellatus* is congruent with the geographic distribution, host fire ant and morphological differentiation of the female ovipositor. Specifically, populations from Buenos Aires (Argentina) that parasitize *S*. *richteri* have an ovipositor with a more laterally pronounced central extension than that of populations from northeastern Argentina and São Paulo (Brazil), which parasitize *S*. *invicta* [8]. This supports the observed paraphyly, in which subtropical *P*. *cultellatus* associated with *S*. *invicta* is more closely related to *P*. *pradei* + *P*. *notocaudatus* + *P*. *disneyi* than to the *P*. *cultellatus* of central eastern Argentina that parasitizes *S*. *richteri*. Possibly, the individuals referred as *P*. *cultellatus* “a”, in Figs 4A and 5, correspond to the original described species, parasitizing *S*. *invicta*, because this sample is geographically closer to the type locality (Rio Negro, Paraná state, Brazil; [68]) than the other Argentine samples studied.

Most of the *P*. *nocens* and *P*. *litoralis* populations that we genetically analyzed seem to belong to a single species according to the delimitation analyses. Both share similar annual and daily activity patterns [23, 67] and host preferences [4]. Alternatively, they could be different species that diverged very recently. The latter hypothesis is supported by the fact that some of the populations that have an overlapped geographic distribution (Fig 4B) showing differences in the ovipositor morphology. Even though both species have a trilobed ovipositor, high resolution SEM imaging (from [8]) shows that *P*. *litoralis* has a group of long setae in the base of the lateral lobes. These are absent in *P*. *nocens* [8], in which the lateral lobes are also more rounded [69]. Whatever scenario is supported, these two species are very closely related and should be reviewed as more evidence becomes available.

The populations of *P*. *nudicornis* studied not only differ in their phylogenetic position, but also in the host fire ant. The population from Goiás, Brazil, parasitizes *S*. *saevissima*, while the populations from Bolivia and Argentina parasitize *S*. *invicta* or *S*. *interrupta* (Fig 3). Being the only species with a bilobulated ovipositor, populations from Goiás (*P*. *nudicornis* “a”) have differences in the female ovipositor compared to the rest of Brazilian populations (e.g. São Paulo flies that parasitizes *S*. *invicta*), which present a more concave plate of the lateral lobes [8].

The samples of nominal *P*. *tricuspis* resolved into two separate, well-supported clades. Populations of *P*. *tricuspis* from Buenos Aires and Córdoba (central Argentina), which parasitize *S*. *richteri*, should be separated from populations of *P*. *tricuspis* that occur elsewhere. In the tree topologies obtained during the phylogenetic and species delimitation analyses, these samples were always separated from the rest of *P*. *tricuspis* and were placed as sister group to *P*. *fowleri* + *P*. *lenkoi* clade. Additional evidence supporting these fly populations as a different species are their geographic distributions in the Pampa ecoregion for the “*P*. *tricuspis* a” (Fig 4F) and their host specificity to *S*. *richteri*. Indeed, members of the other *P*. *tricuspis* clade are mainly found parasitizing *S*. *invicta* and *S*. *interrupta*, but not *S*. *richteri*. In relation to this, [8] mentioned that this switch of host could be related to variations in the shape of the ovipositor, regarding its central extension and membranous filaments, between the populations of São Paulo (Brazil), Santa Fe and those from Buenos Aires (Argentina). The original description of *P*. *tricuspis* places the type locality in La Plata, Argentina [68], corresponding to the populations that we call as *P*. *tricuspis* “a”. Thus, the populations in the other clade should be considered as a new *Pseudacteon* species.

### Phylogenetic relationships and divergence times

The taxon sampling in this study, which includes most of the South American fire ant decapitating fly species of the *Pseudacteon* genus, provides a better phylogenetic resolution than the previous molecular approaches conducted by [20] and [22, 23]. It is worth noting that our analyses revealed both the existence of new (cryptic) species, but also the existence of paraphyly within several current nominal species. For those cases in which morphology is not sufficient to differentiate some species (cryptic species), a more detailed review of the morphological characters (e.g. internal morphology) according to the cryptic lineages reported in this study will be needed.

Several hypotheses regarding the relationships between some *Pseudacteon* species have been raised in the morphological descriptions made by [27] who proposed the closeness of *P*. *affinis* with *P*. *wasmmani*, and of *P*. *dentiger* with *P*. *litoralis* and *P*. *tricuspis*. Our results confirm these associations: although the position of some species (*P*. *litoralis-nocens*, *P*. *calderensis* and *P*. *solenopsidis*) varied between the different phylogenetic reconstruction methods, all of these species were recovered within the same major clade. It is likely that the uncertainty of their topological placement arises from recent divergence times and associated low levels of genetic differentiation, as in their host fire ants.

The genus *Solenopsis* originated in the Neotropical region in the early Oligocene (39.1 Ma, 95% HPD 31.4–47.1) [70]. Even though our estimates are preliminary, the root age of analyzed *Pseudacteon* is close to the Oligocene–Miocene transition, just before the appearance of fire ants. This time period also seems to coincide with the moment when *P*. *convexicauda* and fly species that parasitize South American fire ants of the *S*. *saevissima* species-group were separated. This is also consistent with the origin of *Nylanderia* genus in Oligocene–Miocene transition (~23–24 Ma) [71, 72]; as *P*. *convexicauda* is known to parasitize *N*. *fulva*.

The existence of two main clades in the South American fire ant decapitating flies which diverged about ~19 mya, is intriguing because there are no supported ecological or behavioral patterns that characterize these two clades. Nevertheless, it can be observed that the main clade A tends to be composed of medium to small body size flies (<0.44 mm), while the main clade B has both small and large body size flies (Fig 5). Indeed, species of clade B are the only ones reported parasitizing the larger fire ant species, such as *Solenopsis quinquecuspis* Forel 1913, *Solenopsis weyrauchi* Trager 1991, and *Solenopsis megergates* Trager 1991. Furthermore, the head size of parasitized *S*. *geminata* and *S*. *invicta* workers that have been parasitized is known to be correlated with the body size of their *Pseudacteon* parasitoid species [66, 73]. Therefore, it is likely that difference in body size of the host fire ants could be related to the diversification of both clades during the evolutionary history of these flies. However, no co-phylogenetic analysis could be done, as a well-resolved phylogeny of the fire ants is not currently available.

It is important to remark on the phylogenetic position of *P*. *convexicauda*. This species has been reported using both the tawny crazy ant (*N*. *fulva*) and the fire ant *S*. *saevissima* as host [1, 27]. However, the association with fire ants is very rare [8] and still needs to be confirmed by host rearing observations. In the populations of *P*. *convexicauda* that we surveyed in Argentina, flies were usually found hovering over nests of *N*. *fulva* and attacking their workers, supporting that the assumption this species does not use *S*. *saevissima* species-group as host. This result is congruent with the fact that *P*. *convexicauda* is well differentiated from the rest of the *Pseudacteon* species studied here.

To understand the existence of host switching and/or co-evolutionary relationships between *Pseudacteon* flies and their host fire ants it is necessary to have a well resolved phylogeny of the latter. Fortunately, the phylogenetic relationships within *Solenopsis* fire ants are currently under revision [24] due to the existence of putative cryptic species [74], specifically in the fire ant *S*. *invicta* clade (Dietrich Gotzek, *pers*. *com*.), which contains one of the most detrimental invasive ant species [26].

### Implications for fire ant biological control programs

Historically, the species *P*. *curvatus*, *P*. *litoralis*, *P*. *tricuspis*, *P*. *obtusus*, *P*. *nocens*, and *P*. *cultellatus* have been released to control *S*. *invicta* in the US [12–14]. Our study provides evidence that three of these species harbor cryptic species, or at least genetically well differentiated lineages. The existence of cryptic species could limit the effectiveness of biological control programs and may result in the rejection of potential new agents due to misinterpretation between the fly lineage studied and its host fire ant breadth [19]. Thus, our findings improve the perspective of the range of phenotypic variation and the species boundaries of the taxa, constituting valuable information for natural enemy selection in the biological control program against fire ants. Accordingly, these results help identify candidate lineages with more robust understanding of phylogenetic relationships and evolutionary history. For example, before *P*. *obtusitus* was recognized as a new species (previously identified as the small biotype of *P*. *obtusus*), it was already defined as a different lineage based on molecular evidence at the time it was used for biocontrol [21]. Additionally, when selecting taxa as a biological control agent, it is important to consider that lineages from different geographical regions may differ both genetically and in their bioclimatic affinities [75]. Thus, not only it is important to consider the information about the phylogenetic relationships, but also how these flies partition the host resource along different environmental axes [4, 23].

This work provides information that will allow testing whether the different lineages (putative cryptic species) of the fire ant decapitating flies show differences in their effectiveness as biocontrol agents. Our results should be considered when assessing potential new taxa as possible candidates. Such is the case for populations of *P*. *nocens* from Santiago del Estero, Argentina, which have high levels of host specificity to the biotype of *S*. *invicta* present in that region [76]. In our study, these populations appear as a well-defined group that parasitizes *S*. *invicta* and is placed as a sister group to the *P*. *litoralis* that has been previously used as biocontrol agent [77]. Also, we provide COI (barcode sequences) that may be useful for the molecular identification of males, which are undescribed and relatively unknown yet, because they are poorly attracted to fire ant mounds and therefore rarely collected [8]. Future work is still necessary to better understand the relationships with other species of the *Pseudacteon* genus, including the small *P*. *tricuspis* biotype from the state of Goiás, Brazil (Pesquero *et al*. 2013), those few not included in our study (*P*. *bulbosus*, *P*. *comatus* and *P*. *conicornis*) and those that occur on fire ant species in the *S*. *geminata* species-group in North America. The application of additional methodologies would be useful to determine the robustness of our findings. In particular, matching the lineages of the fire ant *S*. *invicta* species-complex with their corresponding parasitoid fly lineages will surely improve the selection of biocontrol agents against this highly invasive pest ant.

## Supporting information

S1 Table Primers used to amplify the mitochondrial *cytochrome oxidase I* (COI) and the nuclear *wingless* (*wg*) genes. (XLSX)

S2 Table Summary of the ABGD results with or without the outgroups. As input a Kimura two parameters (K2P) distance matrix was used and four different relative gap width values (X) and intraspecific divergence (P) values between 0.001 (0.1%) and 0.1 (10%). († = values in parentheses do not include de outgroups).
(XLSX)

S3 Table Summary of the GMYC results using single and multiple thresholds, and with or without the outgroups. All the analyzes were done using a BEAST ultrametric tree obtained using a strict molecular clock and coalescent constant tree prior. (* = significative values; † = values in parentheses do not include de outgroups, ML = maximum likelihood, LR = likelihood ratio).
(XLSX)

S4 Table Summary of the mPTP results using single and multiple thresholds, and with or without the outgroups. All the analyzes were done using a RAxML best fit tree. († = values in parentheses do not include de outgroups).
(XLSX)

S5 Table *Pseudacteon* species sampled, locality data, ant host and their corresponding GenBank accession numbers. *P*. *obtusus* biotypes are noted as follows: M = medium size, L = large size. Ant host genus abbreviated as: S. = *Solenopsis*; N. = *Nylanderia*.
(XLSX)

S1 Fig ABGD species delimitation output trees. A) including and B) excluding the outgroups. Trees shown correspond to a relative gap width value of 0.9. Numbers at the end of the names correspond to the groups delimited.
(TIF)

S2 Fig GMYC species delimitation output trees including outgroups. A) single thresholds, B) multiple thresholds. Colored lines indicate the cladogenetic events (groups considered as a single species), in the case of multiple thresholds different colors are used in correspondence to each threshold.
(TIF)

S3 Fig GMYC species delimitation output trees excluding outgroups. A) single thresholds, B) multiple thresholds. Colored lines indicate the cladogenetic events (groups considered as a single species), in the case of multiple thresholds different colors are used in correspondence to each threshold.
(TIF)

S4 Fig mPTP species delimitation output trees including outgroups. A) single thresholds, B) multiple thresholds. Colored red lines indicate the cladogenetic events (groups considered as a single species).
(TIF)

S5 Fig mPTP species delimitation output trees excluding outgroups. A) single thresholds, B) multiple thresholds. Colored red lines indicate the cladogenetic events (groups considered as a single species).
(TIF)

S6 Fig Trees obtained using COI + *wg* dataset, only the COI and only the *wg* sequences by each of the selected species/candidate-species. Maximum parsimony strict consensus tree obtained with TNT. Values in branches are boostrap values. A) Maximum parsimony strict consensus trees obtained with TNT, B) Maximum likelihood best trees obtained with RAxML. Values on branches are boostrap values.
(TIF)
