# Correction: A Species delimitation approach to uncover cryptic species in the South American fire ant decapitating flies (Diptera: Phoridae: *Pseudacteon*)

**DOI:** 10.1371/journal.pone.0242952

**Published:** 2020-11-20

**Authors:** Andrés F. Sánchez-Restrepo, Lucila Chifflet, Viviana Andrea Confalonieri, Neil D. Tsutsui, Marcos Antônio Pesquero, Luis Alberto Calcaterra

The sixth author's name is incorrect. The correct name is: Luis Alberto Calcaterra. The correct citation is: Sánchez-Restrepo AF, Chifflet L, Confalonieri VA, Tsutsui ND, Pesquero MA, Calcaterra LA (2020) A Species delimitation approach to uncover cryptic species in the South American fire ant decapitating flies (Diptera: Phoridae: Pseudacteon). PLoS ONE 15(7): e0236086. https://doi.org/10.1371/journal.pone.0236086

The ORCID iD is missing for the sixth author. Please see the author’s ORCID iD here:

- Luis Alberto Calcaterra’s ORCID iD is: 0000-0002-9764-7921 (https://orcid.org/0000-0002-9764-7921).
