# Supplementary figures and images for: A Species delimitation approach to uncover cryptic species in the South American fire ant decapitating flies (Diptera: Phoridae: Pseudacteon)
Source: PLoS One. 2020 Jul 17;15(7):e0236086. doi: 10.1371/journal.pone.0236086 (PMC7367480; doi:10.1371/journal.pone.0236086)

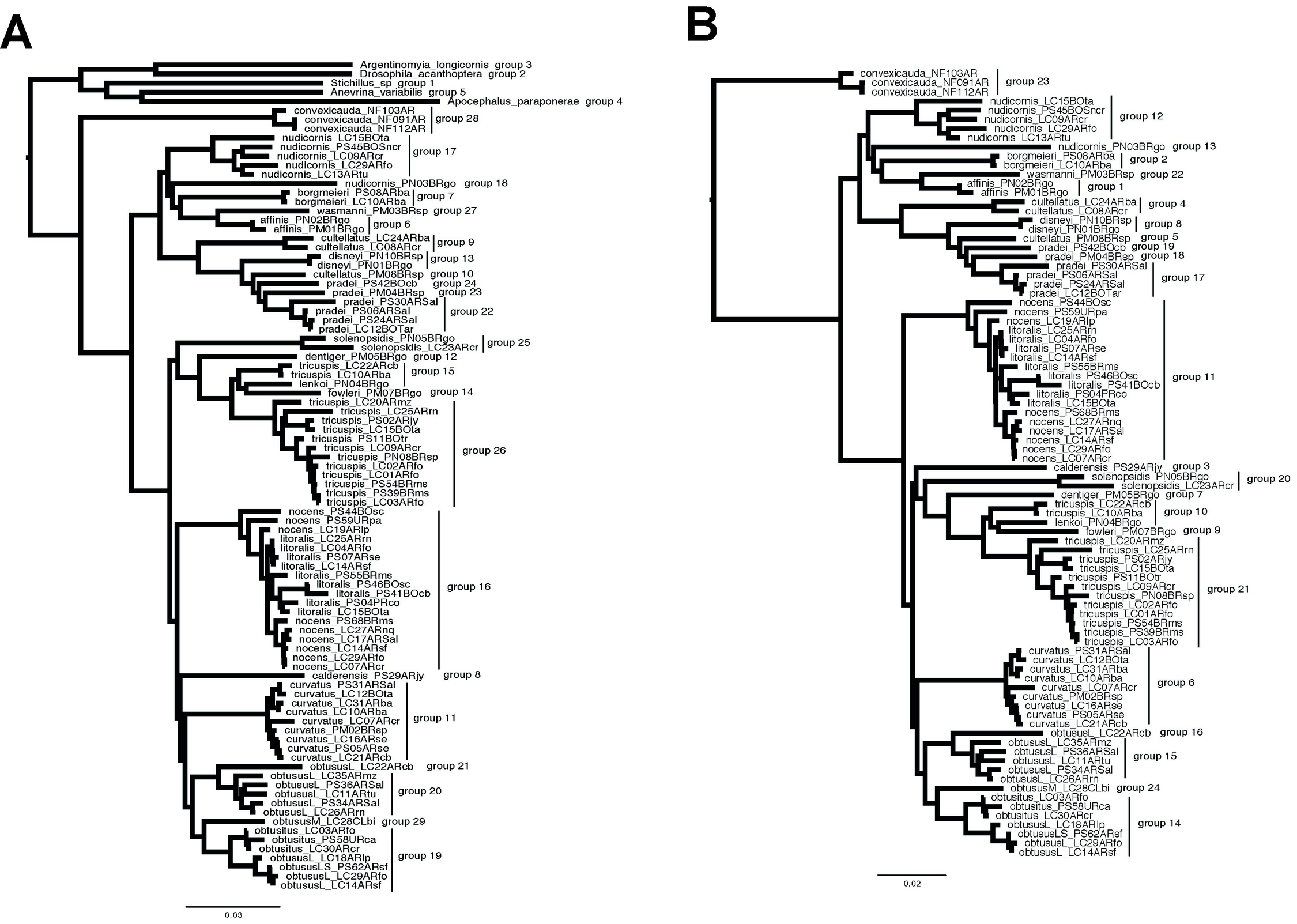

Supplement: S1 Fig — A) including and B) excluding the outgroups. Trees shown correspond to a relative gap width value of 0.9. Numbers at the end of the names correspond to the groups delimited. (TIF) [file pone.0236086.s006.tif]

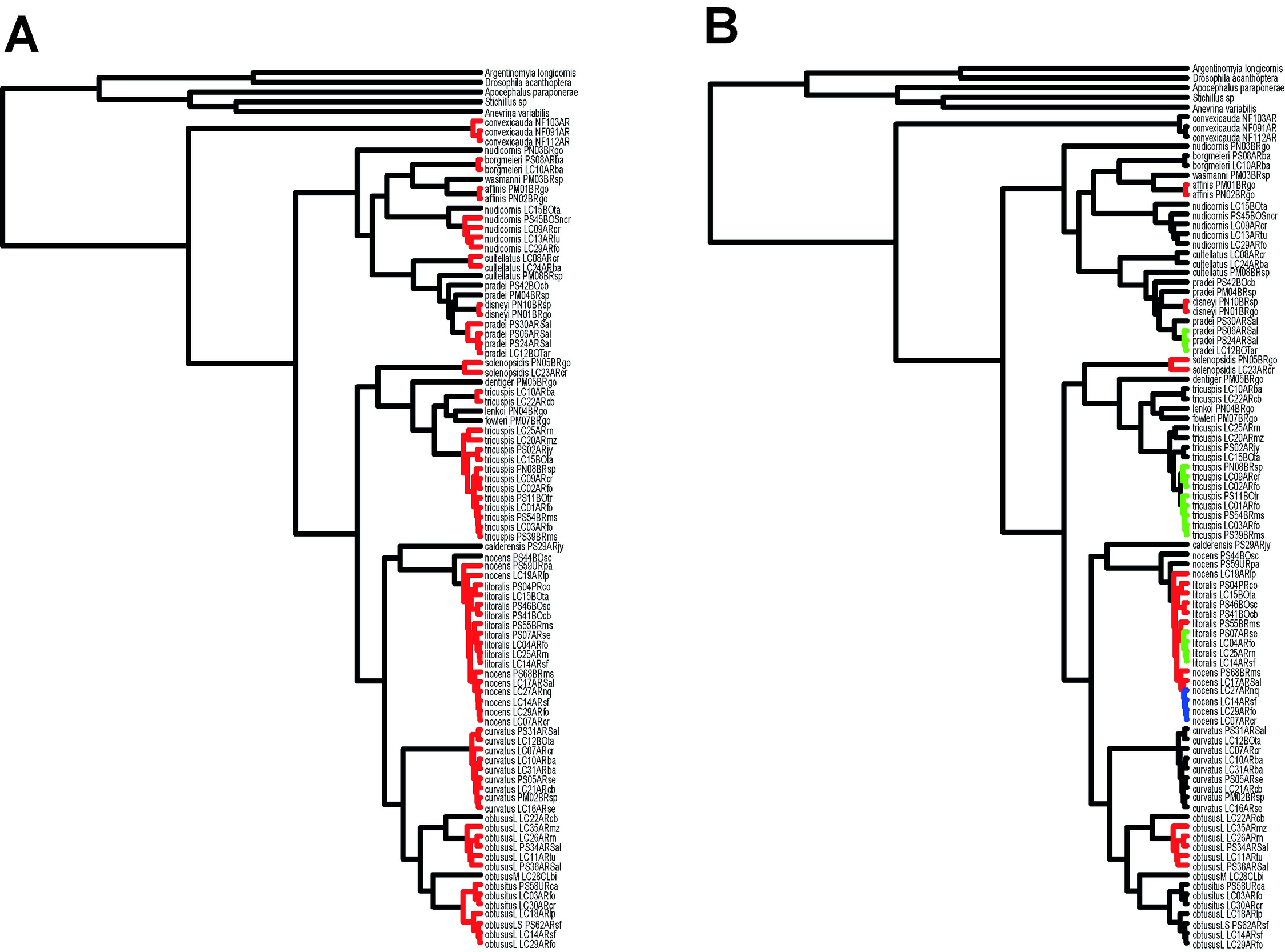

Supplement: S2 Fig — A) single thresholds, B) multiple thresholds. Colored lines indicate the cladogenetic events (groups considered as a single species), in the case of multiple thresholds different colors are used in correspondence to each threshold. (TIF) [file pone.0236086.s007.tif]

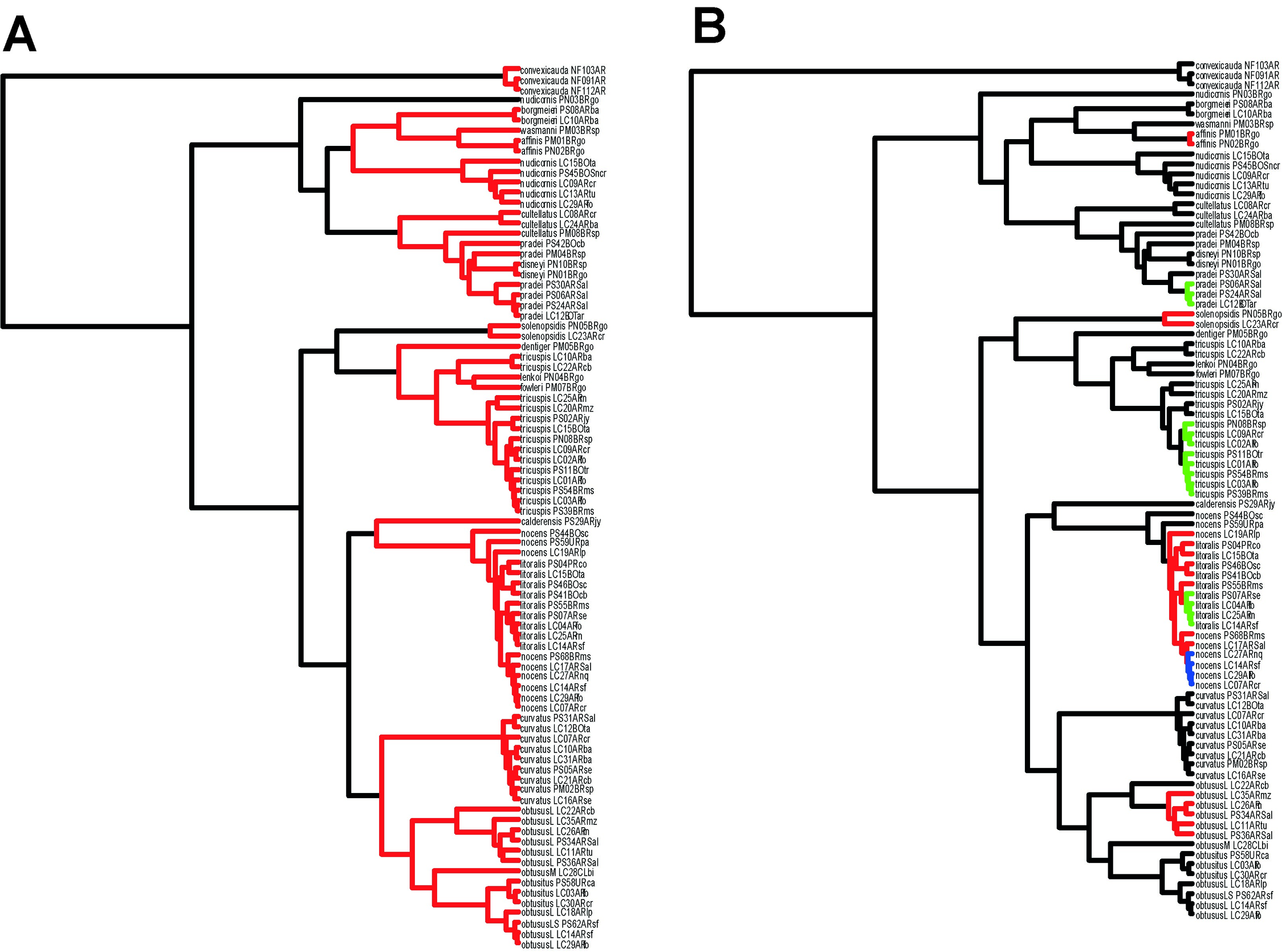

Supplement: S3 Fig — A) single thresholds, B) multiple thresholds. Colored lines indicate the cladogenetic events (groups considered as a single species), in the case of multiple thresholds different colors are used in correspondence to each threshold. (TIF) [file pone.0236086.s008.tif]

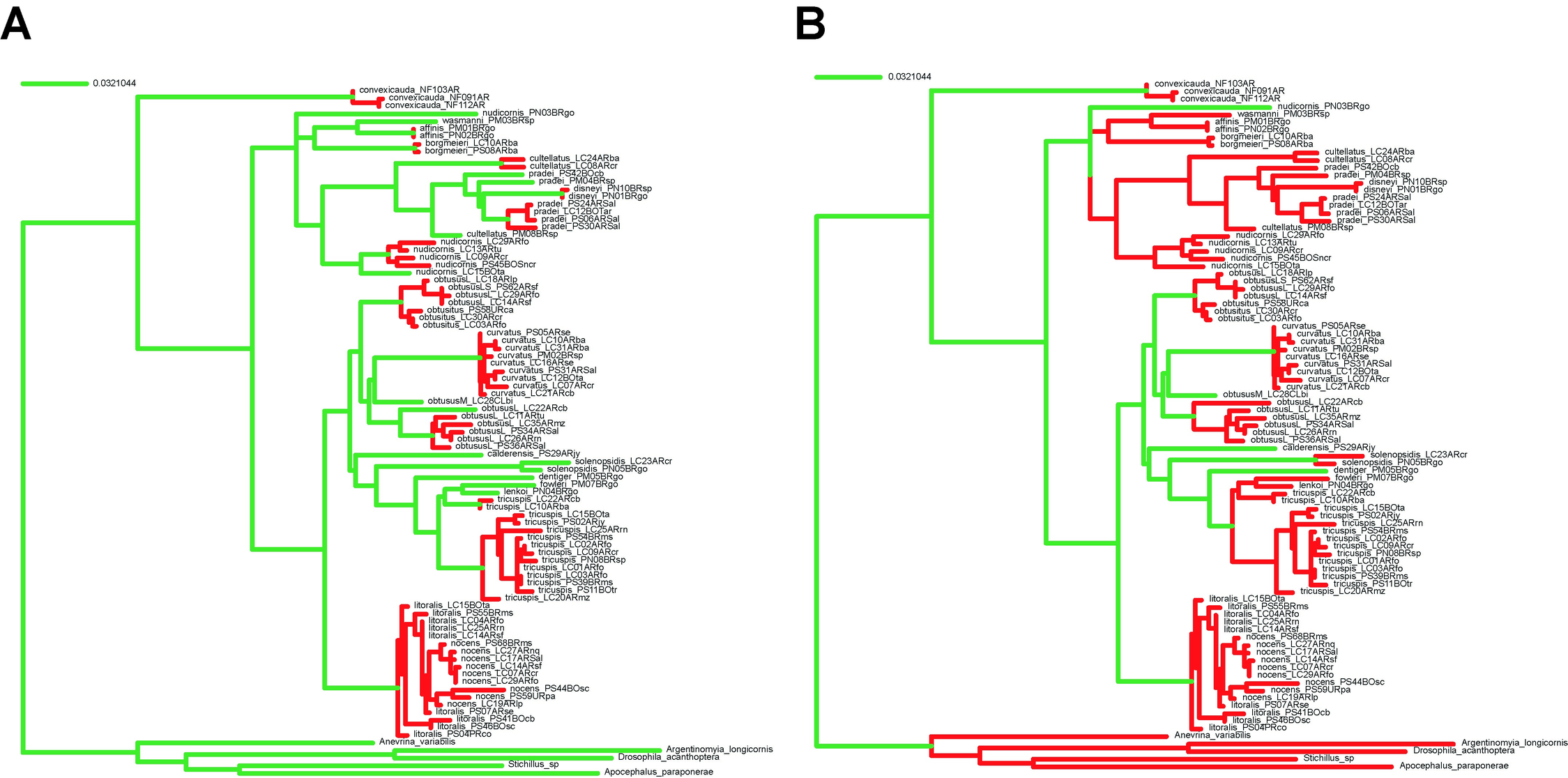

Supplement: S4 Fig — A) single thresholds, B) multiple thresholds. Colored red lines indicate the cladogenetic events (groups considered as a single species). (TIF) [file pone.0236086.s009.tif]

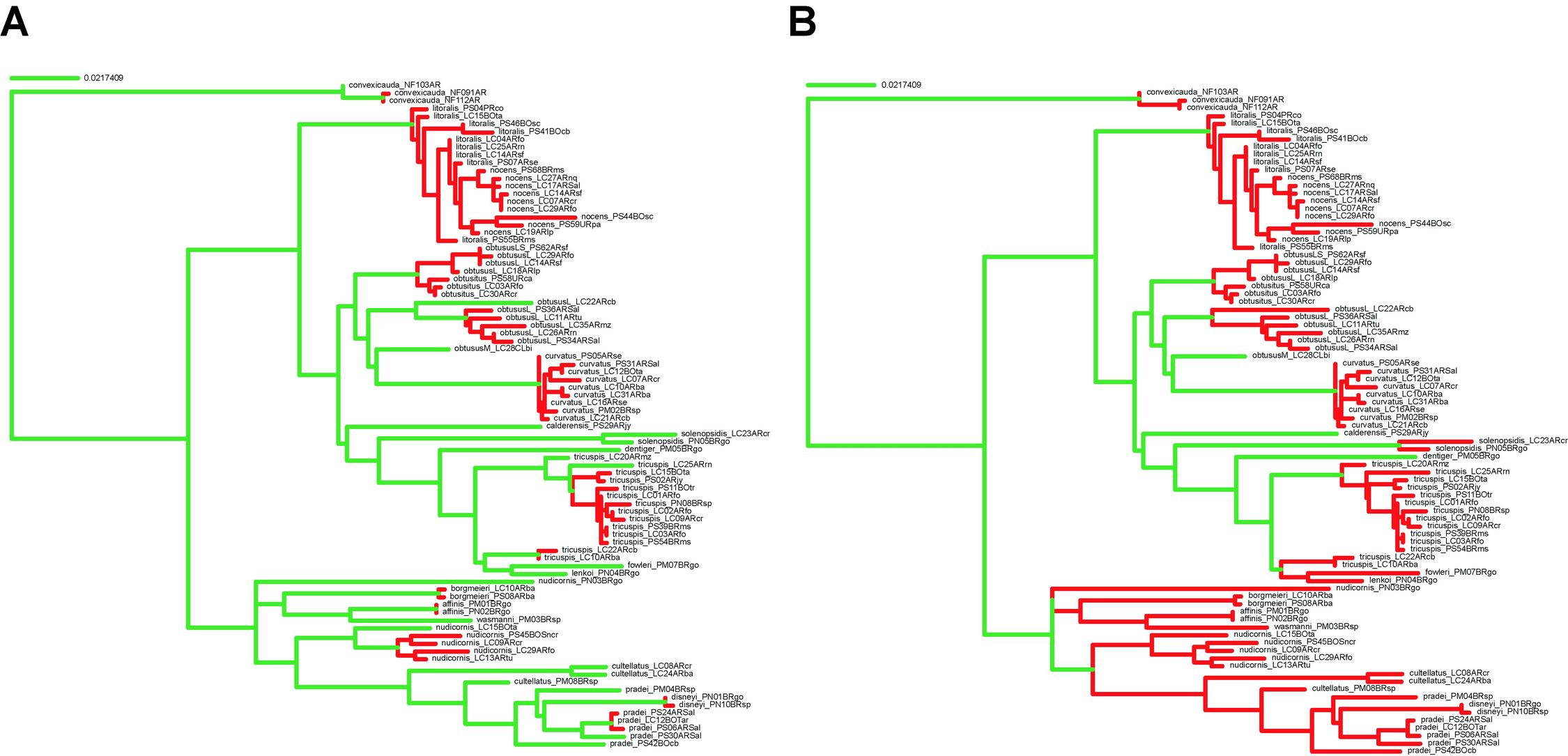

Supplement: S5 Fig — A) single thresholds, B) multiple thresholds. Colored red lines indicate the cladogenetic events (groups considered as a single species). (TIF) [file pone.0236086.s010.tif]

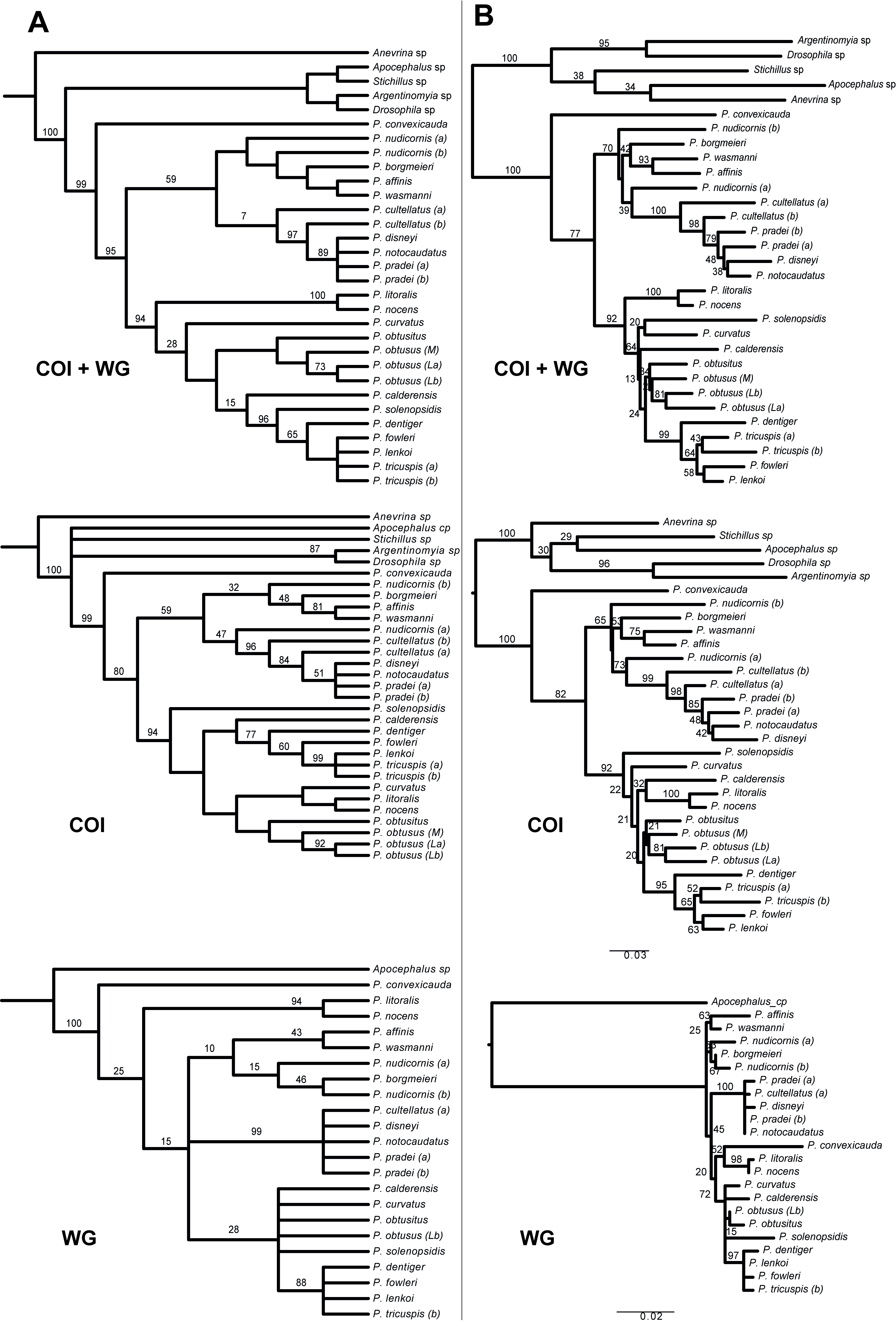

Supplement: S6 Fig — Maximum parsimony strict consensus tree obtained with TNT. Values in branches are boostrap values. A) Maximum parsimony strict consensus trees obtained with TNT, B) Maximum likelihood best trees obtained with RAxML. Values on branches are boostrap values. (TIF) [file pone.0236086.s011.tif]
